# Model Hirano Bodies Protect against Tau-Independent and Tau-Dependent Cell Death Initiated by the Amyloid Precursor Protein Intracellular Domain

**DOI:** 10.1371/journal.pone.0044996

**Published:** 2012-09-18

**Authors:** Matthew Furgerson, Marcus Fechheimer, Ruth Furukawa

**Affiliations:** 1 Department of Biochemistry and Molecular Biology, University of Georgia, Athens, Georgia, United States of America; 2 Department of Cellular Biology, University of Georgia, Athens, Georgia, United States of America; INSERM U894, France

## Abstract

The main pathological hallmarks of Alzheimer’s disease are amyloid-beta plaques and neurofibrillary tangles, which are primarily composed of amyloid precursor protein (APP) and tau, respectively. These proteins and their role in the mechanism of neurodegeneration have been extensively studied. Hirano bodies are a frequently occurring pathology in Alzheimer’s disease as well as other neurodegenerative diseases. However, the physiological role of Hirano bodies in neurodegenerative diseases has yet to be determined. We have established cell culture models to study the role of Hirano bodies in amyloid precursor protein and tau-induced cell death mechanisms. Exogenous expression of APP and either of its c-terminal fragments c31 or Amyloid Precursor Protein Intracellular Domain c58 (AICDc58) enhance cell death. The presence of tau is not required for this enhanced cell death. However, the addition of a hyperphosphorylated tau mimic 352PHPtau significantly increases cell death in the presence of both APP and c31 or AICDc58 alone. The mechanism of cell death induced by APP and its c-terminal fragments and tau was investigated. Fe65, Tip60, p53, and caspases play a role in tau-independent and tau-dependent cell death. In addition, apoptosis was determined to contribute to cell death. The presence of model Hirano bodies protected against cell death, indicating Hirano bodies may play a protective role in neurodegeneration.

## Introduction

Alzheimer’s disease is a growing, worldwide neurodegenerative disease affecting millions of elderly. Alzheimer’s disease (AD) patients experience progressive dementia as a result of severe neurodegeneration. The autopsied brains of AD patients reveal two hallmark neuropathological protein aggregates, amyloid-beta plaques and neurofibrillary tangles (reviewed in [1], [2]) and also have a higher frequency of intracellular F-actin-rich Hirano bodies than age matched normal subjects [2], [3], [4], [5], [6]. Investigating how these pathologies relate to molecular events leading to neurodegeneration is an extensive and often controversial area of research.

The amyloid cascade hypothesis of Alzheimer’s disease [7], [8], [9], [10], [11], [12] and subsequent refinements positing that oligomeric amyloid-beta species are neurotoxic [13] (reviews [14], [15], [16]) have explained a vast amount of the data and pathology in both humans and model systems. Proteolytic processing of the type 1 transmembrane protein amyloid precursor protein (APP) [17], [18], [19] and sequential cleavage of APP by beta- and gamma-secretase releases an extracellular peptide, amyloid-beta, which aggregates to form plaques (reviewed in [20]). Gamma-secretase cleavage of APP also results in the release of a c-terminal intracellular domain, Amyloid Precursor Protein Intracellular Domain (AICD). Additional caspase cleavage results in another smaller c-terminal intracellular peptide, c31 (reviewed in [20]). Although controversial, it is thought that AICD can participate in signaling pathways (reviewed in [21], [22], [23]).

The second pathological hallmark, neurofibrillary tangles, is derived from the aggregation of the microtubule binding protein tau [24], [25]. Tauopathies such as Frontal Temporal Dementias with Parkinsonism (FTDP) are diseases linked to mutations in tau (reviewed in [26], [27]). These diseases exhibit tau pathology, but unlike AD, no amyloid-beta pathology exists. Thus, mutant tau contributes to neurodegeneration without amyloid-beta. Numerous observations suggest a link between the cleavage products of APP and tau in neurodegeneration. Amyloid-beta plaques appear before neurofibrillary tangles in brains during neurodegeneration. Furthermore, reduction of tau levels in a mouse model of Alzheimer’s disease prevented amyloid-beta induced defects [28], [29]. Turning off tau expression in a mouse model of tauopathy rescued memory defects even though cells still contain neurofibrillary tangles [30]. These observations suggests that tau pathology arises downstream of amyloid-beta pathology. However, the exact mechanism, interaction, and timing of these two pathologies remain to be elucidated.

In contrast to the extensive research on APP and tau, very little attention has been given to Hirano bodies, intracellular protein inclusions. They develop in the brain during normal aging, but are highly prevalent in neurodegenerative diseases such as AD, amyotrophic lateral sclerosis, Creutzfeldt-Jakob disease, and some tauopathies [2], [6], [31], [32], [33], [34], [35], [36], [37]. Hirano bodies are highly ordered filamentous actin (F-actin) arranged in a paracrystalline structure [38], [39]. Electron microscopy has shown that the F-actin appears arranged as either a herringbone or crosshatch pattern depending on the plane of section. Due to the lack of a model system, previous research has focused on studying their ultrastructure and identifying their components [3], [33], [40], [41].

Recently, the creation of model Hirano bodies was discovered through the expression of a truncated version of the 34 kDa actin bundling protein. This truncation consists of the carboxyl-terminal region of 34 kDa protein (CT) [42]. Model Hirano bodies induced by CT expression have been successfully created in Dictyostelium, a variety of mammalian cell lines, and in transgenic mice [42], [43], [44]. Model Hirano bodies are indistinguishable from authentic Hirano bodies on an ultrastructural level and contain some of the same protein components including tau and c-terminal fragments of APP [6], [35], [43], [45], [46].

Previous work from our laboratory has shown that model Hirano bodies protect against AICDc58-dependent cell death in H4 cells [47]. This work also showed that model Hirano bodies lowered the transcriptional activation from exogenous AICDc58 [47]. We have extended these studies by investigating the effect of model Hirano bodies on the presence of exogenous APP, AICDc58, and c31. Since tau co-localized with Hirano bodies and tau exhibits pathology in neurodegenerative diseases, we have studied the effect of tau in the presence of APP, AICDc58, and c31 and model Hirano bodies. We provide direct evidence for enhanced cell death between APP/AICDc58, APP/c31 and 352PHPtau, and AICDc58/352PHPtau. We show that these enhanced cell death conditions are partially attributed to activation of apoptotic pathways. Furthermore, we show that model Hirano bodies protect against APP/AICDc58, AICDc58/352PHPtau, APP/c31, and APP/c31/352PHPtau-induced cell death. Understanding the physiological role of Hirano bodies and their interactions with tau and fragments of APP may provide fundamental new insights to understanding Alzheimer’s disease and the presence of Hirano bodies in other neurodegenerative diseases.

## Results

### Model Hirano bodies protect against APP and c31-induced cell death

**Figure 1:**
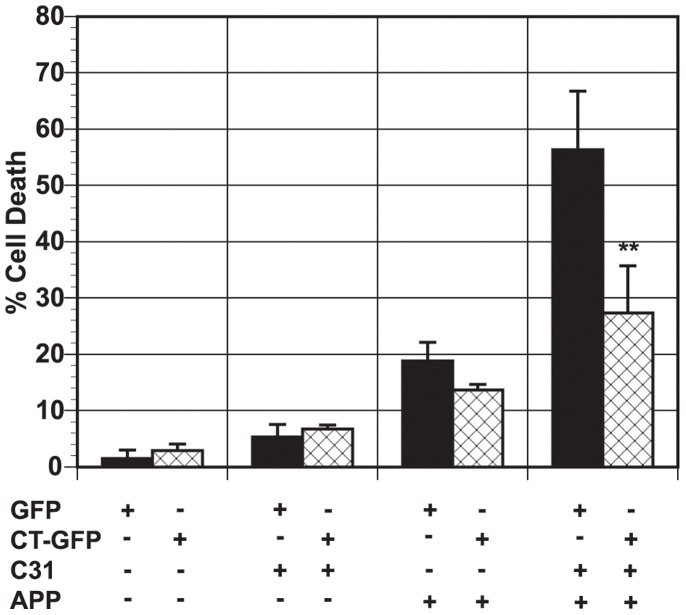
Model Hirano bodies protect against APP and c31-induced cell death in N2A cells. N2A cells were transfected with APP/APPc31 in the presence (CT-GFP, check bars) or absence (GFP, black bars) of model Hirano bodies. Either APP or APPc31-myc causes low amounts of cell death while the presence of both APP/c31 shows an enhanced increase in cell death. Furthermore, model Hirano bodies are able to protect against the enhanced effect of APP/c31-induced cell death (** p<0.01 between cells with and without Hirano bodies). Error bars represent the standard deviation.

It was shown previously that APP enhances the level of cell death induced by c31 [48]. We investigated the effect of Hirano bodies on c31-induced cell death. We transiently expressed APPc31-myc and/or APP to induce cell death in N2A cells as described previously [48], and measured cell death in the presence or absence of model Hirano bodies as shown in Figure 1. Expression of either APP or APPc31-myc alone induced low levels of cell death in the presence or absence of model Hirano bodies. Cells lacking model Hirano bodies and expressing APP/c31 showed a significant increase in cell death as previously reported [48]. In contrast, cells expressing model Hirano bodies and both APP/c31 showed a significant reduction in cell death levels (**p<0.01 compared to cells without model Hirano bodies).

**Figure 2:**
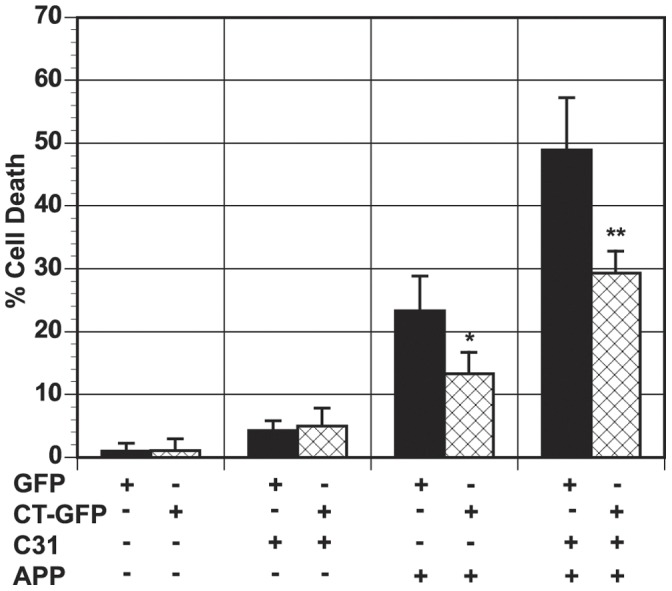
Model Hirano bodies protect against APP and c31-induced cell death (tau-independent) in H4 cells. Cells were transfected with APP/APPc31 in the presence (CT-GFP, check bars) or absence (GFP, black bars) of model Hirano bodies. Expression of either APP or APPc31-myc cause low amounts of cell death while the presence of both APP/c31 caused an enhanced increase in cell death. Furthermore, model Hirano bodies are able to protect against cell death induced by APP (*p<0.05 between cells with and without Hirano bodies) and APP/c31 (** p<0.01 between cells with and without Hirano bodies). Error bars represent the standard deviation.

Since N2A cells contain moderate levels of endogenous tau [49], we investigated whether APP/c31-induced cell death requires the presence of tau. To address this question, we utilized astroglioma derived H4 cells that have very low levels of endogenous tau [49], [50]. Hirano bodies have been proposed to have a glial origin [51] and astrocytes have been shown to be important mediators of cell death induced by beta amyloid and tau [52]. APP and/or c31 were transiently expressed in H4 cells and cell death was measured as shown in Figure 2. Low levels of cell death were observed in the presence of exogenously expressed APPc31-myc as previously observed in N2A cells. Expression of exogenous APP increased the level of cell death in H4 cells similar to the results obtained in N2A cells. In the presence of very low amounts of endogenous tau, model Hirano bodies still protected H4 cells against APP-induced cell death (*p<0.05). In the presence of both exogenous APP and APPc31-myc, enhanced cell death occurred in H4 cells in the presence or absence of model Hirano bodies. The presence of model Hirano bodies significantly lowered the amount of cell death induced by APP/c31 expression (**p<0.01). Cell death pathways of APP and c31 can operate independently of tau. In addition, the presence of model Hirano bodies protect cells from APP/c31-induced cell death.

**Figure 3:**
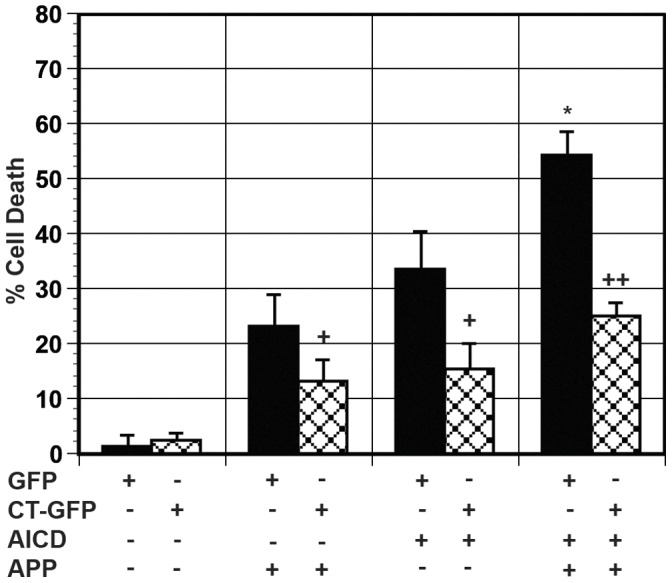
Model Hirano bodies protect against AICDc58 and APP/AICDc58-induced cell death. Cells were transfected with APP and/or AICDc58 in the presence (CT-GFP, check bars) or absence (GFP, black bars) of model Hirano bodies. AICDc58 causes significant cell death in H4 cells in the absence of exogenously expressed APP. Transfection of H4 cells with APP/AICDc58 causes a significant increase in cell death (* p<0.05 in comparison to GFP/AICDc58). The presence of model Hirano bodies significantly protected against AICDc58 and APP-induced cell death (+ p<.05 between with and without model Hirano bodies) and APP/AICDc58-induced cell death (++ p<0.01 between with and without model Hirano bodies). Error bars represent the standard deviation.

Previously, we observed AICDc58-dependent cell death in H4 cells without expression of either exogenous APP or tau [47], [53] and that model Hirano bodies protect against cell death. We investigated whether model Hirano bodies would protect against the enhanced cell death induced by APP/AICDc58 by cotransfection of APPc58-myc plasmid and APP. Expression of either exogenous APPc58-myc alone or APP/AICDc58 caused a significant amount of cell death as shown in Figure 3. The presence of model Hirano bodies decreased the amount of cell death by approximately 50% (+p<0.05). This level of protection is in agreement with previous data [47]. AICDc58-induced cell death was increased approximately 60% in the presence of exogenous APP. The presence of model Hirano bodies significantly reduced APP/AICDc58 cell death (++p<0.01).

#### Model Hirano bodies protect against tau-dependent cell death

**Figure 4:**
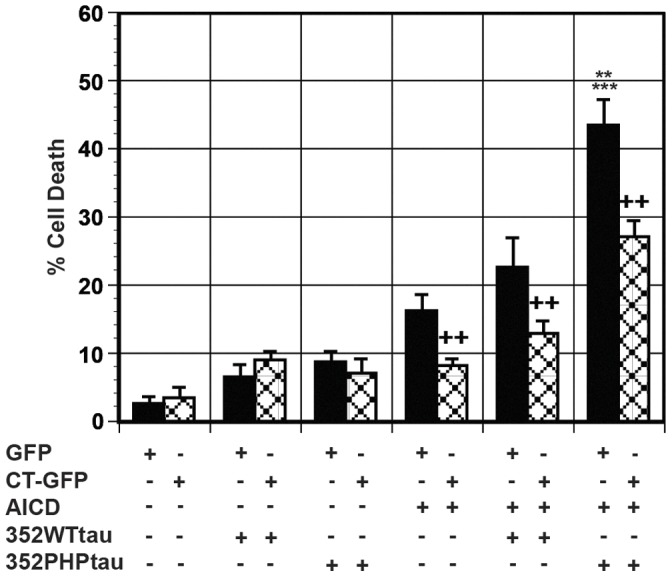
AICDc58 potentiates 352PHPtau, but not 352WTtau-induced cell death. Cells were transfected with AICDc58/352WTtau or AICDc58/352PHPtau in the presence (CT-GFP, check bars) or absence (GFP, black bars) of model Hirano bodies. Co-transfection of H4 cells with GFP and AICDc58/352WTtau seems to result in cell death that approximates GFP/352WTtau plus GFP/AICDc58 cells. Co-transfection of GFP and AICDc58/352PHPtau significantly enhances cell death compared to GFP/AICDc58 only (***p<0.005) or GFP/AICDc58/352WTtau (**p<0.01). The presence of model Hirano bodies significantly protected against AICDc58-, AICDc58/352WTtau-, and AICDc58/352PHPtau-induced cell death (++p<0.01 compared to cells without model Hirano bodies). Error bars represent the standard deviation.

The effect of tau on AICDc58-induced cell death was investigated. Wild type fetal human tau (352 residues, 3R0N) tau (352WTtau) or 352PHPtau fetal human tau (352 residues) with S198, S199, S202, T231, S235, S396, S404, S409, S413, and S422 mutated to glutamate to mimic phosphorylated residues [54] was utilized. Tau phosphorylation has been implicated in neurodegeneration and expression of 352PHPtau has been shown to induce death in a cell culture system at an earlier time point than wild type tau [54], [55]. Exogenous APPc58-myc was transfected alone and with either 352WTtau or 352PHPtau in the presence or absence of Hirano bodies. 352WTtau, like 352PHPtau, causes low levels of cell death when co-expresssed with GFP shown in Figure 4. Expression of exogenous AICDc58 alone causes a significant amount of cell death. AICDc58/352WTtau slightly increases cell death compared to AICDc58 alone. In contrast, 352PHPtau/AICDc58 increases cell death and is significantly higher than both AICDc58-induced cell death (***p<0.005) and AICDc58/352WTtau (**p<0.01). The presence of model Hirano bodies significantly reduced AICDc58, AICDc58/352WTtau, and AICDc58/352PHP-induced cell death (++p<0.01).

**Figure 5:**
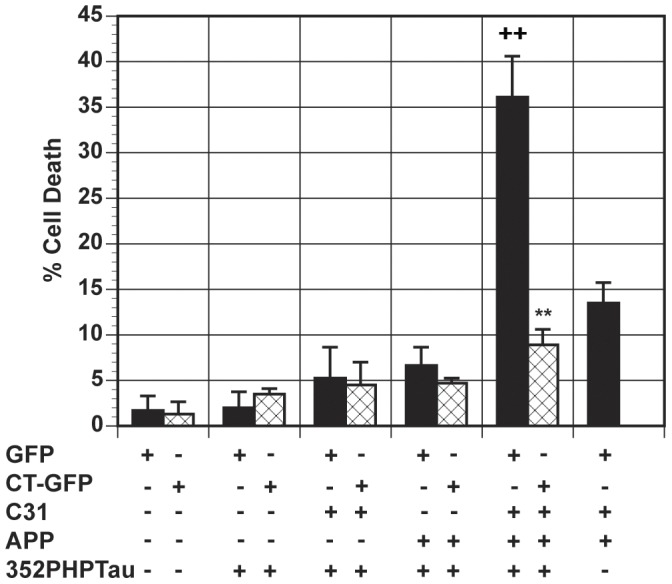
Model Hirano bodies protect against tau-dependent cell death. H4 cells were transfected with APP/c31, and or 353PHPtau in the presence (CT-GFP, check bars) or absence (GFP, black bars) of model Hirano bodies. Transfection of 352PHP tau decreases the amount of exogenous APP (1.5 µg versus 3.0 µg of APP in Figure 2) required to achieve significant enhancement of cell death. Cell death induced by APP/c31/352PHPtau expression is significantly higher than APP/c31 alone (++ p<0.01). Model Hirano bodies protect against tau-dependent cell death (** p<0.01 between with and without model Hirano bodies). Error bars represent the standard deviation.

The effect of tau on APP/c31-induced cell death was investigated. H4 cells were transfected with APP or APP/c31 and 352WTtau or 352PHPtau in the presence or absence of model Hirano bodies. Surprisingly, the 3.0 µg of APP plasmid used to initiate APP and APPc31-myc-induced cell death in H4 cells killed virtually every cell in the presence of APP, 352PHPtau and APPc31-myc (data not shown). Therefore, the amount of APP plasmid was reduced to 1.5 µg in order to achieve a maximum of approximately 50% cell death in the presence of exogenous APPc31-myc and 352PHPtau as shown in Figure 5. A small amount of cell death occurred with expression of 352PHPtau or c31/352PHPtau. At this new concentration of APP plasmid, APP/c31 had significantly lower cell death than with APP/c31/352PHPtau, indicating that the addition of 352PHPtau is responsible for most of the death (++p<0.01 compared to APP/c31). When model Hirano bodies were present, the amount of APP/c31/352PHPtau-induced cell death was significantly reduced (**p<0.01 compared to cells without model Hirano bodies).

#### Both tau-independent and -dependent cell death involve apoptosis

**Figure 6:**
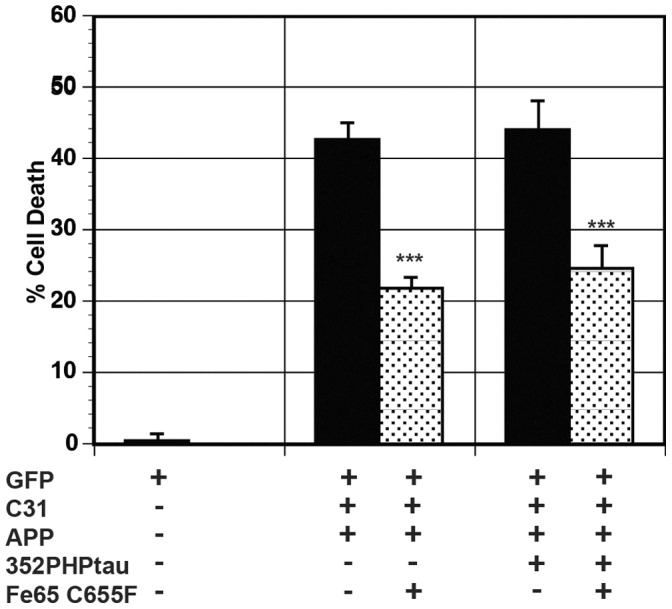
Fe65 contributes to both tau-independent and tau-dependent cell death. Cells were transfected with either APP/c31 or APP/c31/352PHP/tau in the absence (black bars) or presence of C655F Fe65 [56] (dotted bars). The expression of mutant C655F Fe65 significantly reduced both tau-independent and tau-dependent cell death (*** p<0.005). Error bars represent the standard deviation.

**Figure 7:**
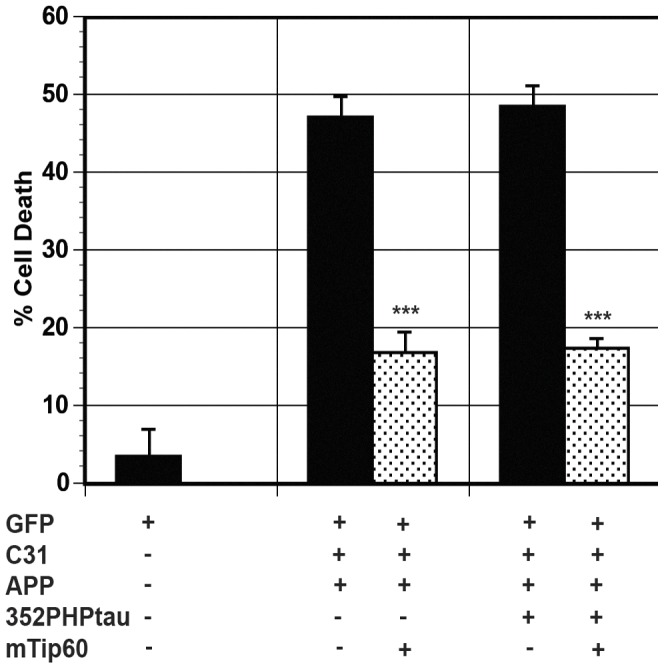
Tip60 contributes to both tau-independent and tau-dependent cell death. Cells were transfected with either APP/c31or APP/c31/352PHPtau in the absence (black bars) or presence (dotted bars) of mutant Q377E G380E Tip60 (mTip60) [53]. The expression of mutant Tip60 significantly reduced both tau-independent and tau-dependent cell death (*** p<0.005). Error bars represent the standard deviation.

To discover how model Hirano bodies protect against tau-independent and tau-dependent cell death, we investigated possible mechanisms of cell death. We previously showed that AICDc58 and Fe65 co-localize with model Hirano bodies and that model Hirano bodies significantly reduced AICDc58-induced cell death and the transcriptional activity mediated by AICDc58, Fe65, and Tip60 [47]. In addition, it has been previously shown that AICDc58-induced cell death involves Tip60 [53]. The contribution of Fe65 and Tip60 in cell death was investigated by utilizing mutations in these proteins. We utilized a previously characterized dominant negative mutation in Fe65, C655F, which abolished its ability to interact with the YENPTY domain of APP or its c-terminal fragments [56]. Exogenous expression of C655F Fe65 significantly reduced both tau-independent (APP/c31) (***p<0.005) and tau–dependent (APP/c31/352PHPtau) cell death (***p<0.005) as shown in Figure 6, implicating Fe65 in both cell death pathways. We utilized a previously characterized dominant negative mutant form of Tip60, Q377E G380E, which abolished the catalytic activity of Tip60 [53]. Expression of Q377E G380E Tip60 (mTip60) significantly reduced both tau-independent (APP/c31) and tau–dependent (APP/c31/352PHPtau) cell death by at least 50% as shown in Figure 7 (***p<0.005). This result indicates a role of Tip60 in both cell death pathways.

**Figure 8:**
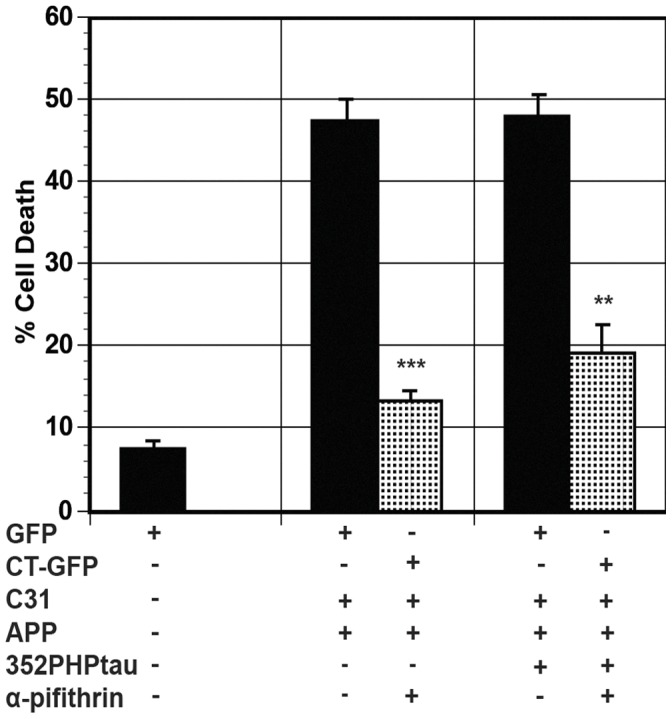
p53 contributes to both tau-independent and tau-dependent cell death. Cells were transfected with either APP/c31 or APP/c31/352PHP/tau in the absence (black bars) or presence (dotted bars) of α-pifithrin. α-pifithrin significantly reduced tau-independent (*** p<0.005) and tau-dependent cell death (** p<0.01). Error bars represent the standard deviation.

It has been reported that AICD and presenilin mediate the upregulation of p53 in cells [57]. Furthermore, Tip60 has been shown to acetylate p53 directly, causing it to initiate transcription of apoptotic genes [58], [59], [60]. We tested the role of p53 in APP/c31-induced cell death in the presence or absence of 352PHPtau using a cell permeable inhibitor of p53, α-pifithrin [61]. Cell death in H4 cells was induced by exogenous expression of APP/c31 or APP/c31/352PHPtau. Cell viability was measured in the presence or absence of α-pifithrin as shown in Figure 8. α-pifithrin significantly reduced both tau-independent (APP/c31) (***p<0.005) and tau-dependent (APP/c31/352PHPtau) cell death (**p<0.01).

**Figure 9:**
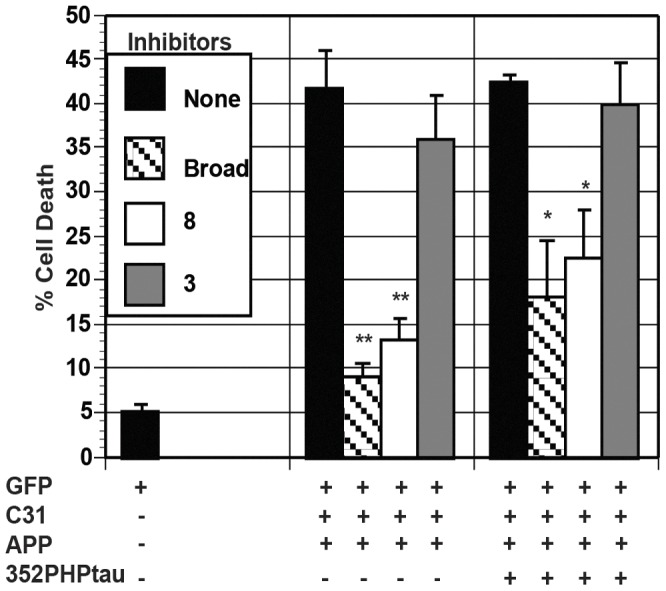
Caspases contribute to both tau-independent and tau-dependent cell death. Cells were transfected with either APP/c31 or APP/c31/352PHP/tau in the absence (black bars) or presence of broad caspase inhibitor (Ac-VAD-CHO; striped bar) or caspase 8 inhibitor (Z-IGTD-FMK; white bars) or caspase 3 inhibitor (Z-DEVD-FMK; grey bars). The broad caspase inhibitor and caspase 8 inhibitor significantly reduced tau-independent (APP/c31) cell death (** p<0.01) and tau-dependent cell death (APP/c31/352PHPtau) (* p<0.05). Caspase 3 inhibitor had no significant effect on either kind of cell death. Error bars represent the standard deviation.

**Figure 10:**
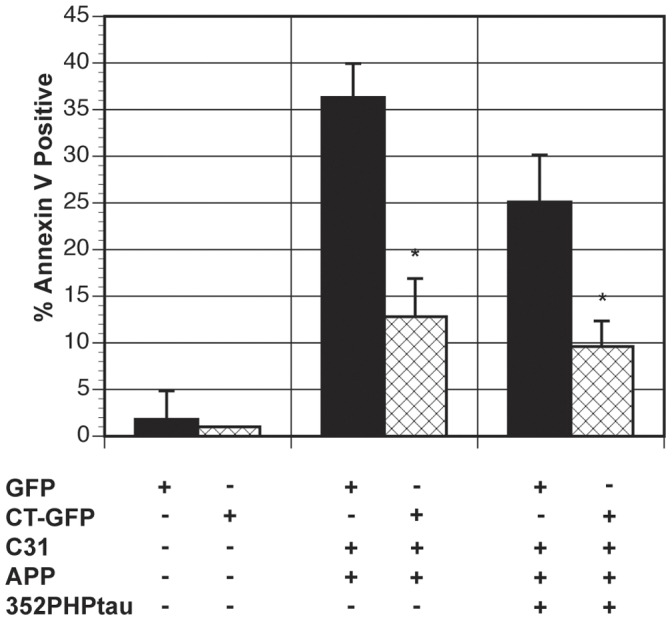
Model Hirano bodies lower tau-dependent and tau-independent apoptosis. H4 cells were transfected with either APP/c31 or APP/c31/352PHP/tau in the absence (GFP-black bars) or presence of model Hirano bodies (CT-GFP-check bars). H4 cells were stained with the early apoptotic marker annexin V. Sytox orange was used as a control for annexin V false positive cells with compromised membranes. Cells transfected with either GFP/APP/c31 (tau-independent) or GFP/APP/c31/352PHPtau (tau-dependent) showed significant levels of annexin V positive staining. The presence of model Hirano bodies lowered the percent of cells that were annexin V positive in both tau-independent and -dependent cell death (* p<0.05 in comparison to cells without model Hirano bodies). Error bars represent the standard deviation.

APP and c31-induced cell death in N2A cells and AICD-induced cell death in H4 cells have been shown to involve caspases [48], [53]. Caspase inhibitors were utilized to investigate whether caspases are involved in APP/c31 and APP/c31/352PHPtau death pathways. Broad-spectrum caspase inhibitor (Ac-VAD-CHO) as well as inhibitors of caspase 8 (Z-IGTD-FMK) and caspase 3 (Z-DEVD-FMK) was utilized in cell death assays in H4 cells as shown in Figure 9. Both the broad and the caspase 8 inhibitors, but not caspase 3 inhibitor, significantly reduced both tau-independent (APP/c31) (**p<0.01) and tau-dependent (APP/c31/352PHPtau) cell death (*p<0.05). Since both p53 and caspases were involved, we hypothesized that apoptosis is likely the mechanism of cell death. To verify that p53 and caspase activation is leading to apoptosis, H4 cells undergoing both tau-independent (APP/c31) and tau-dependent (APP/c31/352PHPtau) cell death were stained with Annexin V. A large population of H4 cells undergoing both tau-independent and tau-dependent cell death were stained by Annexin V, indicating apoptosis is initiated in cell death as shown in Figure 10. H4 cells with model Hirano bodies showed a significant reduction in the amount of Annexin V positive cells compared to H4 cells without model Hirano bodies (*p<0.05) as shown in Figure 10. This observation is consistent with the results above that Fe65, Tip60, caspases, and p53 are involved in both tau-independent and tau-dependent cell death and that model Hirano bodies protect against cell death.

**Figure 11:**
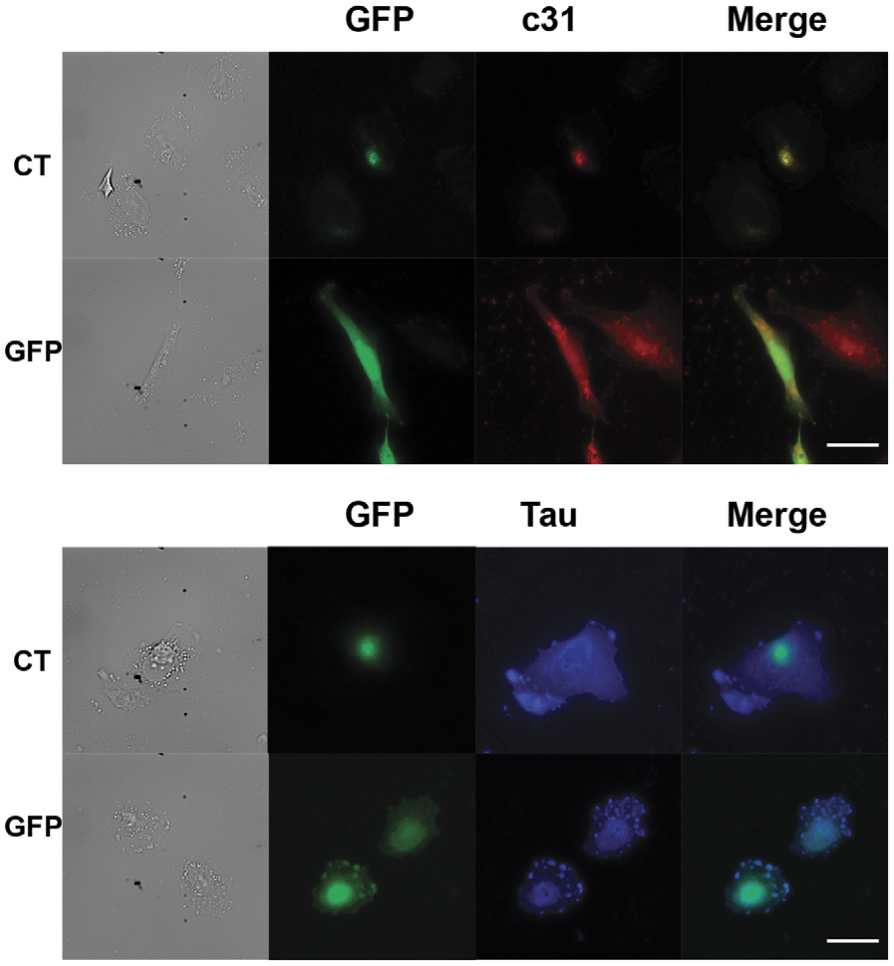
Both 352PHPtau and c31 co-localize with model Hirano bodies. H4 cells were transfected with 352PHPtau or c31, and either CT-GFP to induce model Hirano bodies or GFP as a control. In control cells, tau is predominantly found in the cytoplasm while c31 is evenly diffused throughout the cell. In cells expressing CT-GFP, c31 is primarily found co-localizing with model Hirano bodies. Tau also co-localizes with model Hirano bodies, but its overall distribution is not altered. Scale bar 15 µm.

**Figure 12:**
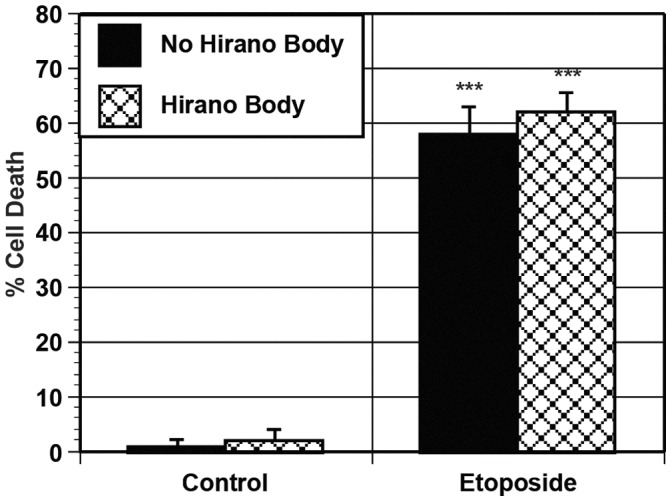
Model Hirano Bodies do not protect against etoposide-induced cell death. H4 cells were transfected with CT-GFP to induce Hirano body formation (check bars) or GFP (black bars) as a control. Expression of CT-GFP or GFP alone caused very low levels of cell death in the absence of etoposide. In the presence of 100 µM etoposide, cell death was significantly increased (***p<0.005). The presence of model Hirano bodies had no impact on etoposide-induced cell death. Error bars represent the standard deviation.

Our results show that Hirano bodies protect against cell death induced by APP/c31 and AICDc58 in the presence or absence of 352PHPtau. Both tau and the c-terminal region of APP have been shown to co-localize with Hirano bodies in post mortem samples as well as in model Hirano bodies in cell culture [6], [35], [43], [47]. This suggests that the protective role of Hirano bodies may be due to sequestering these proteins. To determine if c31 and 352PHPtau co-localize with model Hirano bodies, H4 cells were transfected with c31/352PHPtau and either CT-GFP (CT fused to EGFP) to induce model Hirano bodies or pEGFPN-1 as a control. In GFP control cells, 352PHPtau is predominantly found in the cytoplasm while APPc31-myc is uniformly distributed throughout the cell as shown in Figure 11. Surprisingly, in the presence of model Hirano bodies, APPc31-myc and 352PHPtau are predominantly co-localized with model Hirano bodies. Although APPc31-myc and 352PHPtau co-localize with model Hirano bodies (Figure 11) and cell death induced by these proteins is reduced in the presence of model Hirano bodies, it does not prove that model Hirano bodies reduce cell death by sequestration of these proteins. To address whether the presence of model Hirano bodies protect cells from death specifically, H4 cells expressing GFP or CT-GFP were incubated with and without 100 µM etoposide, a well characterized inducer of apoptosis. Expression of either GFP of CT-GFP in the absence of etoposide results in very low levels of cell death as shown in Figure 12. Cells expressing both GFP and CT-GFP incubated with etoposide had a significant increase in cell death (***p<0.005). The presence of model Hirano bodies had no impact on cell death induced by etoposide. Thus, model Hirano bodies have some specificity protecting against cell death.

## Discussion

The cause of Alzheimer’s disease is unknown [11]. The amyloid cascade hypothesis and subsequent refinement that oligomeric species of abeta contribute to neuronal cell death provide a framework for investigation and explains a wide range of data [14], [16], [62], [63]. AICD is an intracellular product of the processing of APP that produces amyloid-beta. The role of AICD in neurodegeneration is controversial. The lifetime of AICD is short [64], [65] (but tagged versions have a longer lifetime [66], [67], [68]) impeding efforts to identify which cellular pathways it participates in. In spite of this, it has been shown that AICD does activate gene transcription but there are many targets that vary depending on the cell line and strategy used in the investigation (reviewed in [12], [22], [69]). All of these factors lead to controversy and caution in ascribing signaling activity to AICD.

In this study, we have investigated the role of Hirano bodies, intracellular F-actin-rich inclusions, found in a variety of post-mortem neurodegenerative diseases including Alzheimer’s disease [4], [37], [70], [71] and cell death induced by APP, AICDc58 and c31 and tau. Previously, we had shown that the presence of model Hirano bodies reduced transcription of AICDc58 and Fe65 and reduced AICDc58-induced cell death [47]. We have shown that the intracellular c-terminal fragments of APP, AICDc58 and c31 both induce cell death but that c31 requires the presence of APP to achieve significant levels of cell death. In addition, AICDc58-induced death increases in the presence of APP. These results are in agreement with previous results [47], [48], [53], [72], [73], [74]. The presence of model Hirano bodies significantly reduce the level of cell death.

### Transcription and Cell Death

Since the transactivating activity of the APP/Fe65/Tip60 complex has been consistently observed (reviewed in [22], [23], [74], [75]), we utilized mutants in either Fe65 or Tip60 to elucidate whether they play a role in cell death induced by exogenous AICDc58 or APP/c31. Mutations within the active site of Tip60 reduce AICD transcriptional activity and prevent AICD-induced cell death [53], [56], [76]. Our data is consistent with the gene activation activity of this complex since expression of dominant negative mutant Fe65 and Tip60 significantly reduce both APP/c31 and APP/c31/352PHPtau-induced cell death.

Our results disagree with a prior study which showed a mutant form of AICD (Y682A/Y687A) that was suggested not to bind Fe65, was still active in promoting cell death [53]. A possible explanation for this discrepancy is that the AICD mutation (Y682A/Y687A) was not proven to prevent interaction with Fe65 [53]. Furthermore, AICD mutants with either Y682A or Y687A reduce, but do not abolish the interaction of AICD with Fe65. In contrast, the C655F Fe65 mutant used in our study has been shown to prevent this interaction [56]. Thus, all work is consistent with the finding that Fe65 and Tip60 contribute to AICD-induced cell death.

While the mechanism of AICD and tau-dependent cell death is unknown, AICD-induced cell death in the absence of tau involves induction of apoptosis [53]. We have shown that APP/c31 also induces apoptosis. Tip60 has been shown to regulate the specificity of p53, activating transcriptional preference for pro-apoptotic genes [58], [59], [60]. Furthermore, presenilin-dependent release of AICD is reported to enhance activity and expression of p53 to promote neurodegeneration [57]. An additional report shows that Fe65 can either activate or repress target genes, and promote cell death by controlling expression of caspase 4 [77]. Our data is consistent with these findings since our results show that cell death requires both p53 and caspase activation and cells were stained with Annexin V.

While the cause of neurodegenerative diseases is unknown, there are data that suggests that AICD and/or c31 may play a role. AICD levels have been shown to be significantly higher in AD patient brains compared to age matched controls and correlates to levels of phosphorylated tau in these brains [78], [79]. Caspase cleaved APP is also higher in AD brains suggesting that c31 is produced at a much higher rate in AD patients [80]. Additionally, there is evidence that c-terminal fragments of APP could associate with amyloid beta-induced cell death by a mechanism involving amyloid beta binding directly to APP, inducing its cleavage [48], [81]. In addition, AICD expression in mice has been shown to increase levels of insoluble tau and phosphorylated tau, impaired neurogenesis, and promoted neurodegeneration compared to wild type mice [78], [82]. These transgenic mice also show susceptibility to excitotoxicity stressors, which do not affect wild type mice [83], [84]. In contrast, a small subset of the familial mutations found in presenilin 1 have been shown to decrease AICD production, suggesting that AICD might not play a role in Alzheimer’s disease [85]. The mutant presenilins also lower notch intracellular domain levels [85]. However, the gamma secretase complex has over 50 substrates in cells and other signaling pathways may also be affected by these presenilin mutations [86].

In addition, the role of AICD and Fe65 has been investigated in model mice overexpressing these proteins. Transgenic mice have elevated levels of active GSK-3ß, which is also observed with AD patients [87]. These mice also exhibit hyperphosphorylated tau, increased levels of insoluble tau, and neurodegeneration also found in Alzheimer’s patients [78]. Finally, mice with a mutation in the AICD PDAPP(D664A) which cannot be cleaved by caspases and overexpression of Fe65 have reduced levels of seizures to that approaching wild type mice [84], suggesting a role for further proteolytic processing of AICD to c31 in neurodegeneration.

### Interaction of Tau and c-terminal Fragments of APP

The interaction of tau with APP and its proteolytic fragments in the progression of Alzheimer’s disease has been shown in numerous studies (reviewed [14], [62]). However, a direct link between tau and AICD in cell death has not been documented. We have shown for the first time that both AICDc58 as well as APP/c31-induced cell death significantly increases in the presence of 352PHPtau, a mutant form of tau that mimics hyperphosphorylation [54], [55]. The amount of exogenous APP required to induce cell death in the presence of both c31 and 352PHPtau is lower than for APP/c31 alone. Furthermore, the amount of cell death induced by exogenous APP/c31/352PHPtau is greater than that induced by either APP/c31 or 352PHPtau alone, indicating a relationship between APP, c31, and tau in causing cell death. The presence of model Hirano bodies significantly decreased the level of cell death. In contrast, 352WTtau, unlike 352PHPtau did not significantly increase AICDc58-induced cell death. The mechanism underlying the ability of 352PHPtau to significantly increase AICDc58-induced cell death compared to 352WTtau is not clear. It has been shown that phosphorylation of specific residues on tau is required to prime tau for additional phosphorylation [88], [89], [90], [91]. It has also been shown that 352PHPtau becomes phosphorylated in cell cultures [92]. It is possible that the additional phosphorylation of 352PHPtau contributes to the enhanced cell death observed in the presence of either AICDc58 or c31 but the exact mechanism is unknown.

Numerous prior studies support the interaction of APP and tau in progression of Alzheimer’s disease. A link between APP and tau has been supported in whole animal models [28], [29], [93]. Others have shown that cognitive decline in transgenic mice, due to expression of familial disease mutant forms of APP, are ameliorated by deletion of tau [28], [29], [94]. It has recently been shown that amyloid-beta can cause tau hyperacetylation in primary neurons through an unknown mechanism [93], [95]. Acetylation of tau prevents its degradation by competing with ubiquitination, which targets tau to the proteasome. Phosphorylated forms of tau begin to accumulate leading to hyperphosphorylated states [93]. We suggest tau enhances the level of APP/AICDc58 and APP/c31-induced cell death, and could contribute to progression of Alzheimer’s disease.

### Model Hirano bodies protect against cell death

Hirano bodies are found in increased frequency in autopsied brains with neurodegenerative diseases compared to control brains [4], [37], [70], [71]. The physiological role of Hirano bodies is unknown. Development of cell culture and transgenic mouse models of Hirano bodies provide avenues of study that are not available using autopsy and necropsy samples [43], [44] to determine the physiological role of Hirano bodies. Our results obtained using the cell culture model system show that cell death induced by either APP/c31 in the presence or absence of 352PHPtau or APP/AICDc58 is significantly reduced in the presence of model Hirano bodies. Both tau and the c-terminal region of APP have been shown to co-localize with Hirano bodies in post-mortem samples as well as in model Hirano bodies in cell culture [6], [35], [43], [47]. In addition, the APP binding adaptor protein Fe65 has also been shown to co-localize with Hirano bodies in brain and cell cultures [6], [47]. Both c31 and 352PHPtau co-localize with model Hirano bodies, but unlike 352PHPtau, the distribution of c31 changes from diffuse in control samples to concentrated and co-localized with Hirano bodies. The interaction of tau with Hirano bodies could occur through tau and its phosphorylated forms binding directly to F-actin [96]. Other findings in different experimental models suggest that tau promotes the formation of Hirano bodies and that actin promotes tau-dependent toxicity [96], [97]. It is not known if specific isoforms of tau or whether phosphorylation of tau is required for interaction with model Hirano bodies.

We suggest that model Hirano bodies could confer protection against cell death by sequestering c-terminal fragments of APP and possibly tau, preventing them from either participating in signaling pathways which contribute to cell death or gene transcription. Model Hirano bodies can be degraded by either autophagy or through the proteasome pathway [98]. Via these pathways, deleterious proteins bound to the model Hirano bodies are cleared from the cell, possibly conferring protection from cell death. We showed that model Hirano bodies are unable to protect cells against etoposide-induced apoptosis. This lack of protection suggests that Hirano bodies may have a specific role in protection against cell death.

It will be important in the future to determine the effects of model Hirano bodies on the progression of neurodegeneration in animal model systems to determine whether they represent a neuroprotective mechanism.

## Materials and Methods

### Plasmids

pEGFPN-1 was obtained from Clontech. CT-EGFP was previously described [43]. APPc58-myc was generously provided by Dr. Bradley T. Hyman, Harvard Medical School [53]. APP was generously provided by Dr. Thomas C. Südhof, Stanford University School of Medicine [56], [76]. 352tau is fetal human tau (352 residues) and 352PHP-tau is fetal human tau (352 residues) with S198, S199, S202, T231, S235, S396, S404, S409, S413, and S422 mutated to glutamate to mimic phosphorylated residues was generously provided by Dr. Roland Brandt, University of Heidelberg [54]. APPc31-myc was constructed by deleting the coding region of c58-myc from APPc58-myc, inserting the coding sequence of c31 (664–695 of APP) made by PCR amplification using APPc58-myc as a template, and ligating into plasmid that remained from APPc58-myc after deletion of the APPc58-myc coding sequence. Mutagenesis of Tip60 and Fe65 [56], [76] (generously provided by Dr. Thomas C. Südhof, Stanford University School of Medicine) were performed using Stratagene Quickchange mutagenesis kit. DNA sequencing (Genewiz) was performed to confirm sequence fidelity of the Q377E G380E Tip60 and C655F Fe65 mutations and APPc31-myc.

#### Cell Culture and Transfection

All cell lines were grown in DMEM supplemented with 10% fetal bovine serum (FBS) at 37°C and 5% CO_2_. Cells were plated in a 96-well plate (Nunc) or 35 mm dishes approximately 24 hours prior to transfection. Transient transfections were performed using Fugene 6 (Roche Diagnostics) or Lipofectamine LTX with Plus reagent (Invitrogen) according to the manufacturer’s recommendations. Approximately 4 hours after transfection, cells were washed with PBS, resuspended in DMEM plus 10% FBS and grown for approximately 48 hours before cell death assays were performed. N2A mouse neuroblastoma cells (ATCC) were transfected using Fugene 6 with 2.5 µg APPc58-myc, 1 µg APPc31-myc, and 2 µg pEGFPN-1 or CT-EGFP. H4 human astroglioma cells (ATCC) were transfected with Fugene 6 with 3 µg or 1.5 µg APP (tau-dependent and tau-independent conditions, respectively), 1 µg APPc31-myc and either 2 µg pEGFPN-1 or CT-EGFP for the tau-independent assays. For tau-dependent assays, 1 µg 352PHP-tau or 352WTtau was used. For AICDc58 experiments, 1 µg APPc58-myc, 1.5 µg APP, and 1 µg 352PHPtau or 352WTtau were used. Other experiments used 3 µg APP, 3.5 µg APPc58-myc, and 1 µg APPc31myc with either 2 µg of pEGFPN-1 or CT-EGFP. For experiments with Fe65 and Tip60, 1 µg Q377E G380E Tip60 or C655F Fe65 was used. Lipofectamine LTX transfection was used in Figures 4 and 11 with 250 ng of APP, APPc58-myc, 352PHPtau, 352WT tau, pEGFPN-1 or CT-EGFP.

Flow cytometry was used to verify that the presence of multiple plasmids did not affect the level of protein expression (see Figure S1 and Method S1).

Inhibitors used during the cell death assays were added immediately after removal of the Fugene 6 transfection reagent. A final concentration of 20 µM α-pifithrin (Sigma) and 100 µM caspase inhibitors Z-IGTD-FMK (caspase 8), Z-DEVD-FMK (caspase3), and Ac-VAD-CHO (broad caspases) (MP Biomedicals) were used and incubated for 48 hours before cell death assays were performed. A final concentration of 100 µM etoposide (Sigma-Aldrich) was added approximately 24 hours after the transfection reagent was removed. After two hours, cells were washed with PBS and resuspended in DMEM plus 10% FBS and incubated at 37°C with 5% CO_2_ for 16 hours prior to cell death assays.

#### Cell Death Assays

Cells were incubated with 9 nM Sytox Orange nucleic acid dye (Invitrogen) and 264 µM Hoechst 33258 at 37°C and 5% CO_2_ for 10 minutes prior to microscopy. Fluorescence and visible cell images were obtained using a Zeiss IM-35 epi-fluorescence microscope equipped with a CCD 300-T-RC camera (Dage MTI) controlled by Scion Image software. GFP and Sytox Orange fluorescence and phase images were superimposed in Photoshop and live and dead cells were counted (see Figure S2 for representative images). Since N2A and H4 cell lines have low transfection efficiencies (∼15%), the presence of EGFP or CT-EGFP was used to indicate transfected cells. A minimum of 50 cells was counted per sample and each condition was sampled at least three independent times. The mean value is plotted in the histograms with error bars representing the standard deviation. Statistics were performed using the Student’s T-test.

#### Annexin V Staining

Approximately 24 hours after transfection, cells were washed with annexin binding buffer (10 mM HEPES, 140 mM NaCl, 2.5 mM CaCl_2_, pH 7.4) and stained with 5 µL Pacific blue-conjugated annexin V (Invitrogen) for 30 minutes at 37°C and 5% CO_2_. Cells were washed once with annexin V binding buffer, re-suspended in annexin V binding buffer with 9 nM Sytox orange, and incubated at 37°C and 5% CO_2_ for 15 minutes prior to assessment by fluorescence microscopy (see Figure S2 for representative images). The fraction of apoptotic cells were scored as GFP positive cells that were also Annexin V positive divided by the total number of GFP positive cells. Sytox orange was utilized to eliminate Annexin V positive cells that had a compromised membrane. A minimum of 50 cells was counted per sample and each condition was sampled at least three independent times. The mean value is plotted in the histograms with error bars representing the standard deviation. Statistics were performed using the Student’s T-test.

#### Immunofluorescence

Approximately 24 hours prior to transfection, cells were plated onto glass coverslips. Cells were cotransfected with 1.5 µg each of pEGFPN-1 or CT-GFP and 352PHPtau/APPc31-myc using 3.5 µL Lipofectamine LTX and 1.0 µL Plus reagent according to the manufacturer’s instructions (Invitrogen). Approximately 4 hours after transfection, cells were washed with PBS, resuspended in DMEM with 10% FBS. Approximately 24 hours after transfection, the cells were washed in PBS and fixed for 10 minutes in 3.7% formaldehyde in PBS and 1.0 mM EGTA (pH 7.0). Cells were permeablized for 5 minutes in 0.1% Triton X-100 in PBS and 1 mM EGTA (pH 7.0). Cells were washed three times in PBS and blocked for 1 hour with 10.0% BSA in PBS. Cells were washed three times in PBS and incubated with mouse anti-myc 1∶1000 (Cell Signaling) and rabbit anti-FLAG 1∶100 (Sigma) primary antibodies for 1 hour. After three washes in PBS, cells were incubated with Alexa 350-labeled goat anti-rabbit 1∶200 (Invitrogen) and TRITC-labeled goat anti-mouse 1∶700 (Sigma) secondary antibodies for 1 hour. Cells were washed three times in PBS and mounted with Crystal Mount (Biomeda). Images were obtained using a Deltavision microscope and subjected to deconvolution using Applied Precision software.

## Supporting Information

Figure S1 **Cell death and GFP expression levels are not affected by varying amounts of DNA during co-transfection of multiple plasmids.** H4 cells were transfected with pEGFPN-1 alone or with varying amounts of pcDNA3.1 to replicate co-transfection conditions with 1, 2, or 4 plasmids. Using flow cytometry, it was determined that the median fluorescence intensity of GFP in samples replicating co-transfection of 2 and 4 plasmids were not significantly different from GFP only transfected cells. Additionally, under these conditions cell death levels remained low, indicating that the cell death in our other experiments is not due to an increase in total DNA transfected. Error bar represents the coefficient of variation.
(TIF)

Figure S2 **Representative images of the cell death quantitation**. Live cells transfected with (A) GFP, (B) CT-GFP, GFP/APP/c31 stained with sytox orange (C) or (D) annexin V and Sytox Orange to mark apoptotic cells and rule out compromised membranes, respectively. Green  =  GFP, Blue  =  Hoescht 33258 (A and B), annexin V (D). Yellow  =  GFP and sytox orange. Arrows in C indicate nuclei stained with Sytox Orange. Scale bar  = 20 µm.
(TIF)

Method S1 **Flow cytometry.**
(DOCX)
