# Supplementary figures and images for: Model Hirano Bodies Protect against Tau-Independent and Tau-Dependent Cell Death Initiated by the Amyloid Precursor Protein Intracellular Domain
Source: PLoS One. 2012 Sep 18;7(9):e44996. doi: 10.1371/journal.pone.0044996 (PMC3445605; doi:10.1371/journal.pone.0044996)

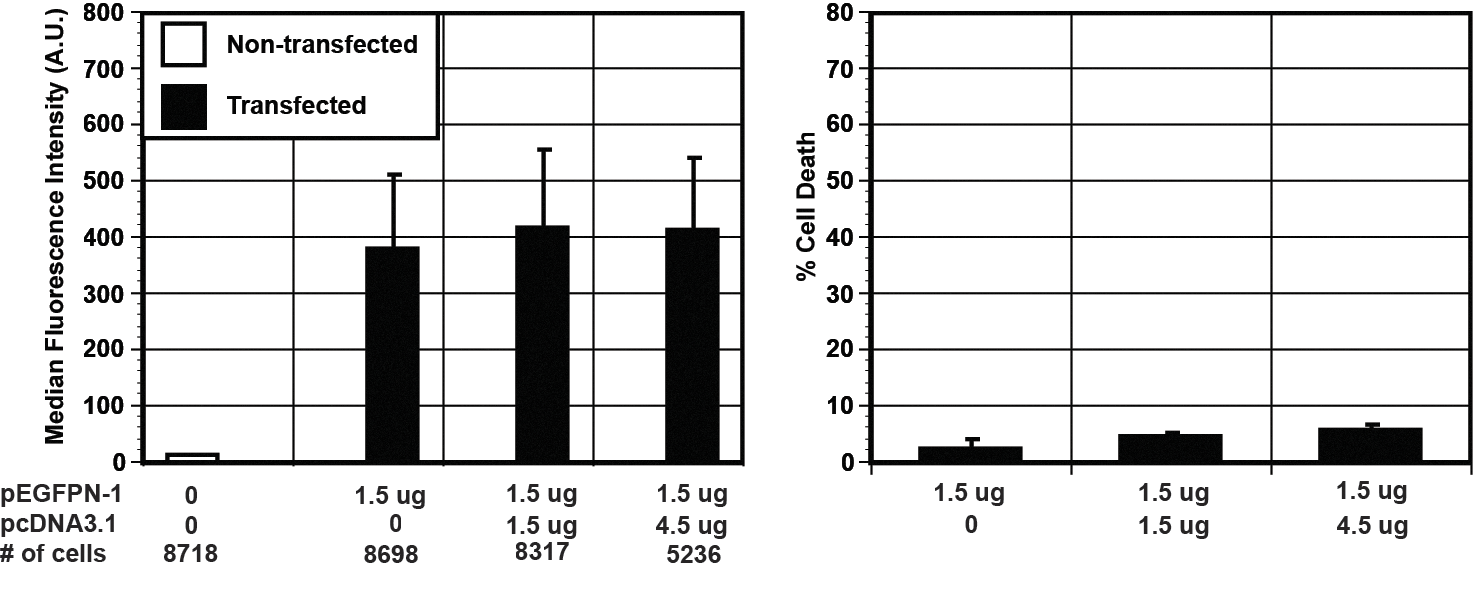

Supplement: Figure S1 — Cell death and GFP expression levels are not affected by varying amounts of DNA during co-transfection of multiple plasmids. H4 cells were transfected with pEGFPN-1 alone or with varying amounts of pcDNA3.1 to replicate co-transfection conditions with 1, 2, or 4 plasmids. Using flow cytometry, it was determined that the median fluorescence intensity of GFP in samples replicating co-transfection of 2 and 4 plasmids were not significantly different from GFP only transfected cells. Additionally, under these conditions cell death levels remained low, indicating that the cell death in our other experiments is not due to an increase in total DNA transfected. Error bar represents the coefficient of variation. (TIF) [file pone.0044996.s001.tif]

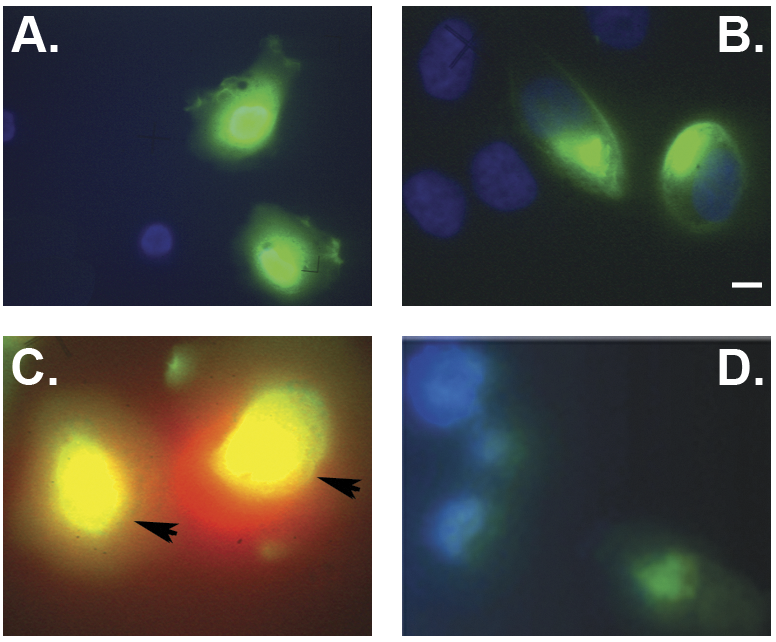

Supplement: Figure S2 — Representative images of the cell death quantitation. Live cells transfected with (A) GFP, (B) CT-GFP, GFP/APP/c31 stained with sytox orange (C) or (D) annexin V and Sytox Orange to mark apoptotic cells and rule out compromised membranes, respectively. Green = GFP, Blue = Hoescht 33258 (A and B), annexin V (D). Yellow = GFP and sytox orange. Arrows in C indicate nuclei stained with Sytox Orange. Scale bar = 20 µm. (TIF) [file pone.0044996.s002.tif]
