# Supplementary material for: Model Hirano Bodies Protect against Tau-Independent and Tau-Dependent Cell Death Initiated by the Amyloid Precursor Protein Intracellular Domain
Source: PLoS One. 2012 Sep 18;7(9):e44996. doi: 10.1371/journal.pone.0044996 (PMC3445605; doi:10.1371/journal.pone.0044996)
Supplement: Method S1 — Flow cytometry. (DOCX) [file pone.0044996.s003.docx]

**Method S1: Flow Cytometry**

100,000 H4 cells were plated in 35 mm dishes approximately 24 hours prior to transfection.  Cells were transfected with 1.50 μg of pEGFPN-1 and a range of pcDNA3.1 (Invitrogen) using 1.5 μL LipofectAMINE LTX and 1.0 μL Plus reagent in serum free DMEM according to the manufacturer’s instructions. Four hours after transfection, cells were washed with PBS, resuspended in DMEM with 10% FBS.  Approximately 24 hours after the transfection reagent was removed, flow cytometry was performed using a Beckman Coulter's Hyper-cyan flow cytometer and data was analyzed using FlowJo software.
